# One-way edge modes in a photonic crystal of semiconductor at terahertz frequencies

**DOI:** 10.1038/s41598-018-26395-4

**Published:** 2018-05-25

**Authors:** Lingjuan He, Qian Shen, Jie Xu, Yun You, Tianbao Yu, Linfang Shen, Xiaohua Deng

**Affiliations:** 1 Institute of Space Science and Technology, Nanchang University, Nanchang, 330031 China; 2 Department of Physics, Nanchang University, Nanchang, 330031 China

## Abstract

Electromagnetic edge mode in a photonic crystal (PhC), which is a square array of semiconductor rods in air, is theoretically investigated for terahertz frequencies. In the PhC, gyroelectric anisotropy is introduced in the semiconductor rods by applying an external magnetic field and consequently, a degeneracy point, at which two dispersion surfaces intersect, is lifted and a new band gap is created. The edge mode sustained by the PhC possesses the character of one-way propagation, and it even can be immune to backscattering at large defect on the wavelength scale and 90° sharp bend. The properties of the one-way mode are closely dependent on the cladding layer structure of the PhC.

## Introduction

One-way electromagnetic (EM) edge modes were first proposed by Raghu and Haldan as analogues of quantum Hall edge states^1–3^. Such modes are allowed to propagate in only one direction and can be immune to backscattering at imperfections or bends. Recently, one-way EM edge modes have attracted much attention since they provide a basic mechanism for realizing new kinds of optical components, which are not possible using traditional reciprocal EM modes^4–6^. One-way EM modes can be sustained by the edges of photonic crystals (PhCs)^7^ made of magneto-optical (MO) materials^8–11^. Wang and his colleagues demonstrated (both theoretically and experimentally) the existence of one-way EM edge mode at microwave frequencies by using two-dimensional (2D) PhCs made of yttrium-iron-garnet (YIG)^8,9^. Another type of one-way EM edge modes was also proposed later, and it was realized by using surface magnetoplasmons (SMPs)^12,13^. These one-way modes have been intensively investigated more recently^14–19^. SMPs are surface plasmons generally sustained by the interface between a dielectric and plasmonic material, and they can exhibit one-way propagation in the presence of external dc magnetic field^14,15^.

SMPs in a magnetized plasmonic material become nonreciprocal, thus their asymptotic frequency differs in the forward and backward directions^13^. Consequently, SMPs can only propagate in one direction within the interval between the two different asymptotic frequencies. Evidently, the frequency range of one-way SMPs is closely dependent on the plasma frequency of the material. More importantly, as surface waves, the frequency range of one-way SMPs must lie within the band gaps of the material itself^15^. In contrast, one-way EM edge modes in PhCs are supported based on the PhC band gap instead of the material band gap. As the frequencies of band gaps in a PhC generally scale as the inverse of the lattice constant^7^, the frequency range of one-way EM edge mode in PhCs can be flexibly tailored by properly choosing the lattice constant. This also offers the possibility for one-way EM edge modes to lie within a (frequency) window of low material loss. Therefore, it is meaningful to investigate one-way EM edge modes in PhCs in terahertz regime. To our knowledge, the terahertz one-way edge modes in PhCs have not been reported yet. In this paper, we will show that one-way EM edge mode can be supported by a PhC made of magnetized semiconductor at terahertz frequencies and exhibit some interesting behaviours.

## Physical Model

**Figure 1:**
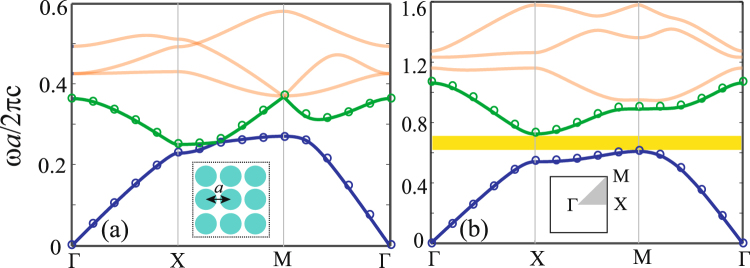
Band structures for the PhCs made of InSb (**a**) without and (**b**) with external magnetic field. The open circles represent the results (for the first two bands) calculated using the finite element method. The inset in (a) is a schematic of the PhC, and the inset in (b) is the first Brillouin zone. The radius of the InSb rods is *r* = 0.46*a*. The external magnetic field in (b) is *B*_0_ = 0.9 T (i.e., *ω*_*c*_ = 0.9*ω*_*p*_).

The photonic crystal under our consideration consists of a square array of semiconductor rods in air, as shown in the inset of Fig. 1(a). The radius of the semiconductor rods is 0.46*a*, where *a* is the lattice constant. An external dc magnetic field is applied along the semiconductor rods (in the –*z* direction), thus the rods become gyroelectric anisotropic, with the permittivity tensor taking the form^13,15^

1 $${\overleftrightarrow{\varepsilon }}_{s}={\varepsilon }_{\infty }\,[\begin{array}{ccc}{\varepsilon }_{1} & -i{\varepsilon }_{2} & 0\\ i{\varepsilon }_{2} & {\varepsilon }_{1} & 0\\ 0 & 0 & {\varepsilon }_{3}\end{array}],$$

with

$$\begin{array}{rcl}{\varepsilon }_{1} & = & 1-\frac{{{\omega }_{p}}^{2}}{{\omega }^{2}-{{\omega }_{c}}^{2}},\\ {\varepsilon }_{2} & = & \frac{{{\omega }_{p}}^{2}{\omega }_{c}}{\omega \,({\omega }^{2}-{{\omega }_{c}}^{2})},\\ {\varepsilon }_{3} & = & 1-\frac{{{\omega }_{p}}^{2}}{{\omega }^{2}},\end{array}$$

where *ω* is the angular frequency, *ω*_*p*_ is the plasma frequency of the semiconductor, *ω*_*c*_ = *eB*_0_/m^*^ (*e* and m^*^ are the charge and effective mass of the electron, respectively) is the electron cyclotron frequency, and *ε*_∞_ is the high-frequency (relative) permittivity of the semiconductor. In Eq. (1), the semiconductor is assumed to be lossless. The material loss will be taken into account later when we examine the robustness of the edge mode character. For the magnetized semiconductor, the dispersion relation of bulk modes is $k=\sqrt{{\varepsilon }_{{\rm{v}}}}(\omega /c)$ for the *H* polarization, where *ε*_v_ is the Voigt permittivity defined by ${\varepsilon }_{{\rm{v}}}={\varepsilon }_{\infty }({\varepsilon }_{1}-{\varepsilon }_{2}^{2}/{\varepsilon }_{1})$, and it becomes $k=\sqrt{{\varepsilon }_{\infty }{\varepsilon }_{3}}(\omega /c)$ for the *E* polarization, on which the external magnetic field has no influence. In this paper, the semiconductor is assumed to be InSb with *ε*_∞_ = 15.68 as an example^15,20^. The plasma frequency is *f*_*p*_ = 2 THz and *ω*_*p*_ = 2*π f*_*p*_. *ω*_*c*_ is set to be 0.9*ω*_*p*_ (corresponding to *B*_0_ = 0.9 T). At the frequency *f* = 3.1 THz, the tensor elements in Eq. (1) are *ε*_1_ = 0.3721, *ε*_2_ = 0.3646, and *ε*_3_ = 0.5838. Correspondingly, the Voigt permittivity *ε*_v_ is equal to 0.2321.

In the case without external magnetic field, i.e., *B*_0_ = 0, InSb is isotropic and its relative permittivity is 9.15 at *f* = 3.1 THz. By using the plane-wave expansion (PWE) method^21^, we calculated the band structure of the proposed PhC (for the *H* polarization) for the case of *B*_0_ = 0 (i.e., *ω*_*c*_ = 0), and the obtained results are plotted in Fig. 1(a). It is seen that no band gap exists in the PhC. The first two dispersion bands intersect at a single point in the Brillouin zone. However, this degeneracy is lifted when an external magnetic field of *B*_0_ = 0.9 T is applied in the PhC, and a band gap occurs between the first two bands, as shown in Fig. 1(b). The band gap ranges from the frequency 0.606 (2*πc*/*a*) to 0.717 (2*πc*/*a*), whose bandwidth is about 16% of the central frequency of 0.662 (2*πc*/*a*). The band gap created by the external magnetic field provides a basis for constructing one-way edge mode. To examine the obtained results, the PhC bands for the two cases with *B*_0_ = 0 and 0.9 T are also calculated by using the finite element method (FEM), and the results for the first two bands are plotted as open circles in Fig. 1(a,b). The results from the two numerical methods are almost the same. In the numerical calculations with the both methods, the tensor elements in Eq. (1) are set to be constants, which correspond to the values at a desired frequency. Here, this frequency is taken to be *f* = 3.1 THz as an example. To ensure the central frequency of the band gap in Fig. 1(b) to lie at *f* = 3.1 THz, the lattice constant of the PhC should be *a* = 61 *μ*m for consistency. Compared with Fig. 1(a), the bands in Fig. 1(b) are shifted up by the external magnetic field. In the case of *B*_0_ = 0.9 T, the Voigt permittivity of the anisotropic InSb is 0.23 (at 3.1 THz), which is much smaller than the (relative) permittivity of 9.15 for the isotropic InSb (with *B*_0_ = 0). Correspondingly, the effective refractive index of the PhC, which is equal to the square root of the averaged permittivity in the unit cell, is found to be 2.53 for *B*_0_ = 0 and 0.70 for *B*_0_ = 0.9 T. The slope of the first band at the Γ point is inversely proportional to the effective refractive index, and this explains why the PhC bands shift up in the presence of the external magnetic field.

**Figure 2:**
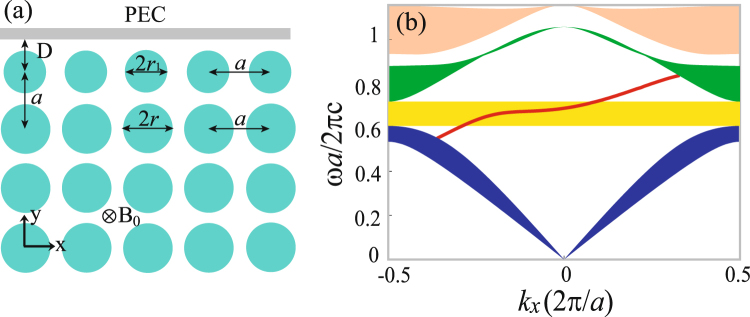
(**a**) Schematic of a PhC system for guiding robust one-way edge mode. The radius (*r*_1_) of the InSb rods at the outmost row may be different from that (*r*) in the PhC. (**b**) Dispersion curve of the edge mode. The shaded rectangular area indicates the band gap of the PhC. Other three shaded areas represent the projected bands of the PhC along Γ − *X* direction. The parameters of the PhC are the same as in Fig. 1(b). *D* = 0.6*a* and *r*_1_ = *r* = 0.46*a*.

The system for guiding one-way edge mode is illustrated in Fig. 2(a). In order to make the one-way mode immune to backscattering, the cladding air on the PhC is terminated by a metal slab, which is at a distance of *D* from the centers of the InSb rods at the outmost row. To investigate the dependence of the one-way mode on the cladding structure, the radius of the InSb rods at the outmost row is allowed to vary and denoted by *r*_1_. Thus, the cladding layer of the PhC is comprised of a metal slab, air layer of the thickness (*D* − *r*_1_), and a row of InSb rods with the radius *r*_1_ in the case if *r*_1_ ≠ *r*. By using the finite element method, we numerically solved for the edge mode through solving Maxwell’s equations in the super cell of the guiding system. The (Floquet) periodic boundary condition (characterized by the Bloch wavevector *k*_*x*_) is employed in the *x* direction, and the super cell is terminated by scattering boundary condition from below. In the calculation, the metal slab is assumed to be perfect electrical conductor (PEC), and this is a valid approximation for the terahertz regime. Figure 2(b) shows the dispersion curve for the edge mode, where *r*_1_ = *r* = 0.46*a* and *D* = 0.6*a* (correspondingly the thickness of the air layer is equal to 0.14*a*). As an edge mode, the dispersion curve is terminated by the boundaries of bulk-mode zones in the PhC. Obviously, the slope of the dispersion curve is always positive, therefore the edge mode only propagate forward. This one-way edge mode covers a (frequency) region larger than the PhC band gap, which is created by the external magnetic field. Interestingly, in the lower part of the one-way region where *k* < 0, the one-way edge mode corresponds to a backward wave, whose phase velocity is negative while the group velocity (corrresponding to the band slope) is positive^22^. Note that the energy flow of the edge mode is in the same direction as the group velocity. Here, it should be indicated that only within the band gap of the PhC, this one-way edge mode can be immune to backscattering. Our numerical calculation also shows that one-way edge mode only propagate backward when the external magnetic field is reversed.

## Robust One-Way Propagation

**Figure 3:**
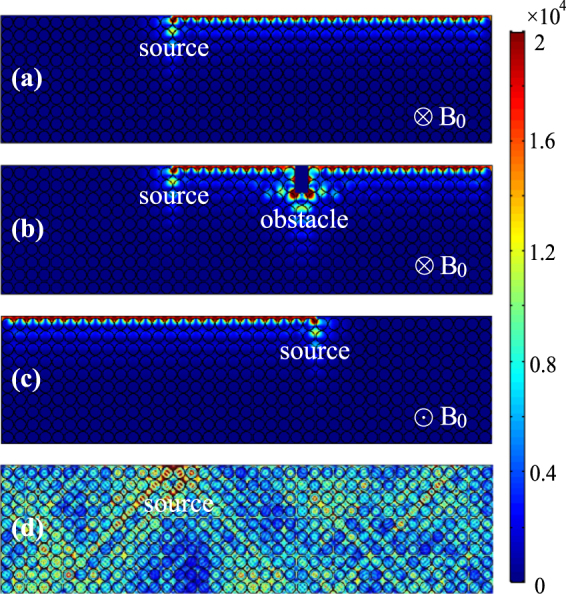
Simulated electric field amplitudes in the PhC systems with different external magnetic fields. (**a**) *B*_0_ = 0.9 T, (**b**) *B*_0_ = 0.9 T but with an obstacle, (**c**) *B*_0_ = −0.9 T, and (**d**) *B*_0_ = 0. The obstacle in (b) is a metal slab of sizes 0.97*a* × 2*a* (i.e., 0.6*λ* × 1.26*λ*). In all cases, the source is a linear magnetic current located in the air layer and the frequency is 3.1 THz. The other parameters of the PhC systems are the same as in Fig. 2.

The edge mode should exhibit robust one-way propagation (ROWP) in the PhC band gap, because no other mode exists there in the system. To verify the robustness of the one-way edge mode, we performed a simulation of wave transmission in the guiding system by using the finite element method. In the simulation, a linear magnetic current was placed in the air layer to excite wave, and the operation frequency is 3.1 THz, which corresponds to the normalized frequency *ωa*/2*πc* = 0.63. The computation domain is properly terminated and scattering boundary conditions are employed on its left, right, and lower sides. The simulated electric field amplitudes are shown in Fig. 3(a). It is seen that an edge mode is excited in the PhC and it only travels forward, which agrees well with our analysis above. To examine the robustness of one-way edge mode, we insert a large obstacle in the guiding system to block the one-way propagating wave. The obstacle is a rectangular metal column with sizes of 0.97*a* × 2*a* (i.e., 0.6*λ* × 1.26*λ*). The simulated results are plotted in Fig. 3(b). As expected, when the excited wave reaches the obstacle, it goes around it and continues to travel forward along the PhC edge. We also performed the simulations for other cases when the external magnetic field in Fig. 3(a) is reversed or removed. As expected, the one-way mode propagates backward when the external magnetic field is reversed, as shown in Fig. 3(c). In the case when *B*_0_ = 0, the semiconductor material becomes isotropic in the PhC and the operation frequency is not again located in its band gap. As seen in Fig. 3(d), waves excited by the magnetic current transmit into the PhC in this case, and radiating fields occur almost everywhere in the PhC.

**Figure 4:**
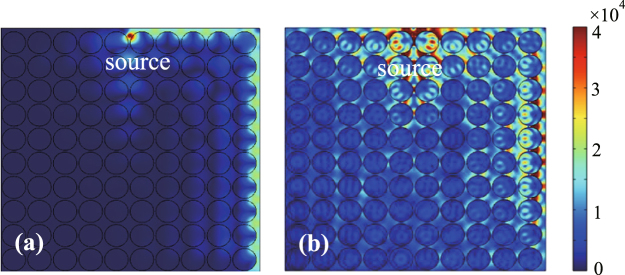
Simulated electric field amplitudes in a 90° bent guiding systems with (**a**) *B*_0_ = 0.9 T and (**b**) *B*_0_ = 0. In (b), edge mode cannot be sustained by the PhC where the external magnetic field is removed. The other parameters of the systems are the same as in Fig. 2.

We further simulated the wave transmission in a 90° bent guiding system. For this case, the scattering boundary conditions are employed at the left and lower (output) ends of the bent guiding system. The simulated electric field amplitudes are plotted in Fig. 4(a). Evidently, when the one-way propagating wave reaches the corner of the system, it completely goes through the sharp bend and continues to travel downward. For comparison, the simulation for the case when *B*_0_ = 0 was also performed and the obtained results are plotted in Fig. 4(b). In this case, the radiating fields present everywhere in the PhC and they are stronger close to the upper and right edges of the PhC, since the radiating fields are reflected there by the PEC boundaries. Note that the left and lower sides of the computation domain correspond to the termination with the scattering boundary conditions. In all simulations above, the materials in the system are lossless, and the transmission rate of the one-way edge mode is found to be perfect (i.e., 100%) at the output ends in Fig. 4(a) as well as in Fig. 3(a–c). The above simulations show that the one-way edge mode in the proposed system is immune to backscattering at the large obstacle on the wavelength scale and 90° sharp bend.

**Figure 5:**
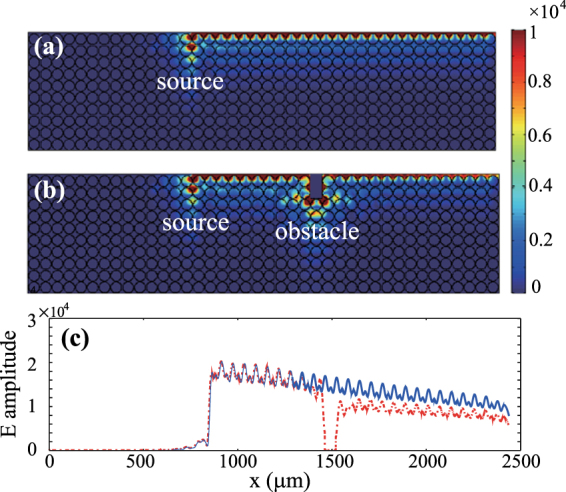
Simulated electric field amplitudes in the lossy systems (**a**) without and (**b**) with obstacle. (**c**) Electric field amplitudes on the central line of the air layer in the system. The solid and dotted lines correspond to the relevant results in (a,b), respectively. The loss parameter is *ν* = 10^−3^*ω*_*p*_, and the other parameters are the same as in Fig. 3(a,b).

As a dispersive medium, the InSb material is inherently lossy. For the lossy InSb, the expressions for *ε*_1_ and *ε*_2_ in Eq. (1) become^15^

2 $$\begin{array}{rcl}{\varepsilon }_{1} & = & 1-\frac{(\omega +i\nu ){\omega }_{p}^{2}}{\omega [{(\omega +i\nu )}^{2}-{\omega }_{c}^{2}]},\\ {\varepsilon }_{2} & = & \frac{{\omega }_{c}{\omega }_{p}^{2}}{\omega [{(\omega +i\nu )}^{2}-{\omega }_{c}^{2}]},\end{array}$$

where the loss parameter *ν* corresponds to the electron scattering frequency. It is known that the band structure of photonic crystal (for real-wavevector/complex-frequency modes satisfying the periodic boundary conditions) usually remains unaffected in the presence of the material loss^23–25^. To examine the influence of the material loss on the edge mode, we performed the simulation of wave transmission once more for the cases of Fig. 3(a,b) but with material loss. As an numerical example, we take *ν* = 10^−3^*ω*_*p*_ for InSb. The simulated electric field amplitudes for the two lossy cases (with and without the obstacle) are plotted in Fig. 5. The situations in Fig. 5 are similar to those in Fig. 3. So the material loss cannot change the character of robust one-way propagation for the edge mode. However, the field amplitude of the one-way edge mode gradually decreases along the propagation length due to the energy dissipation in the InSb rods, as shown in Fig. 5(c), where the solid and dotted lines correspond to the electric-field amplitudes on the central line of the air layer for the cases without and with obstacle, respectively. The transmission (power) rate is also calculated for the propagation distance of 25*a* (i.e., 15.7*λ*) in the simulation. The transmission rate is 34.7% for the case without obstacle and 18.5% for the case with the obstacle. In the latter case, local electric fields around the obstacle are highly enhanced, so is the material absorption loss there. One-way modes can be used for the rigorous trapping of light energy^26^, but the inherent material losses would hamper the practical applications. Fortunately, the material losses can be compensated by introducing optical gain^27,28^.

## Influence of the Cladding Layer

**Figure 6:**
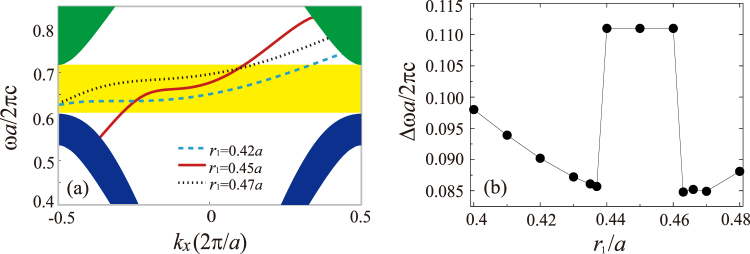
(**a**) Dispersion curves of the edge mode for the different radii *r*_1_ = 0.42*a*, 0.45*a*, and 0.47*a*. (**b**) ROWP bandwidth as a function of the radius *r*_1_. The other parameters are the same as those in Fig. 2.

The influence of the cladding layer of the PhC system on the edge mode is interesting^29^. We first consider the outmost row of InSb rods, and various values of *r*_1_ are analyzed, but the rod centers remain unchange. The parameter *D* is kept to be 0.6*a* for all cases. Our numerical analysis shows that the one-way propagation of the edge mode is preserved when *r*_1_ varies, as illustrated in Fig. 6(a), where the dispersion curves of the edge mode for the different radii *r*_1_ = 0.42*a*, 0.45*a*, and 0.47*a* are plotted. However, the radius *r*_1_ can affect the ROWP bandwidth of the edge mode. Note that robust one-way edge mode must lie within the band gap of the PhC. Figure 6(b) shows the dependence of the ROWP bandwidth of the edge mode on the radius *r*_1_. In the range from 0.44*a* to 0.46*a*, the one-way edge mode can cover the whole band gap of the PhC, and the ROWP bandwidth is always equal to the band-gap width. Outside this range, the ROWP bandwidth abruptly drops, and for *r*_1_ > *r* (where *r* = 0.46*a*), it is always less than the band-gap width.

**Figure 7:**
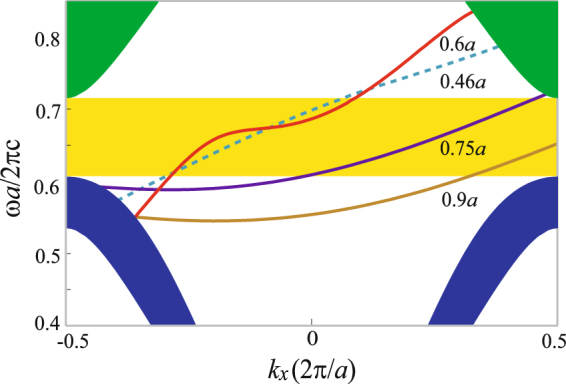
Dispersion curves of the edge mode for *D* = 0.46*a*, 0.6*a*, 0.75*a*, and 0.9*a*. The other parameters are the same as in Fig. 2.

We next investigate the influence of another cladding layer parameter *D* on the edge mode. In this situation, the parameter of *r*_1_ is kept to be 0.46*a*, i.e., *r*_1_ = *r*, thus the variation of *D* only leads to the change of the thickness of the air layer between the PhC and metal slab. Different values of *D* = 0.46*a*, 0.6*a*, 0.75*a*, and 0.9*a* are analyzed. For the four cases, the thickness of the air layer is 0, 0.14*a*, 0.29*a*, and 0.44*a*, respectively. The calculated results are plotted in Fig. 7. Within the PhC band gap, there exists an edge mode for all cases, and it has the character of one-way propagation there. Obviously, the ROWP bandwith of the edge mode is equal to the band-gap width for the three cases of *D* = 0.46*a*, 0.6*a*, and 0.75*a*, and it is reduced in the case of 0.9*a*. Furthermore, our numerical analysis shows that when *D* ≥ 1.004*a*, the edge mode completely vanishes in the PhC band gap. Therefore, the thickness of the air layer should be smaller than a certain value for achieving a broadband one-way edge mode.

## Conclusion

In summary, we have theoretically studied the edge mode in a PhC made of gyroelectric semiconductor at terahertz frequencies. This edge mode is guided based on a band gap which is created by the external magnetic field applied in the PhC. The edge mode exhibits one-way propagation, and its propagation direction can be reversed by reversing the external magnetic field. The property of the one-way edge mode is closely dependent on the cladding layer of the PhC. It has been numerically demonstrated that the one-way edge mode can be immune to backscattering at large obstacle on the wavelength scale and 90° sharp bend. So the proposed one-way edge mode may provide an approach for realizing new kinds of terahertz optical components.
